# Prediction of low cardiac output syndrome in patients following cardiac surgery using machine learning

**DOI:** 10.3389/fmed.2022.973147

**Published:** 2022-08-24

**Authors:** Liang Hong, Huan Xu, Chonglin Ge, Hong Tao, Xiao Shen, Xiaochun Song, Donghai Guan, Cui Zhang

**Affiliations:** ^1^Cardiovascular Intensive Care Unit, Department of Critical Care Medicine, Nanjing First Hospital, Nanjing Medical University, Nanjing, China; ^2^College of Computer Science and Technology, Nanjing University of Aeronautics and Astronautics, Nanjing, China

**Keywords:** cardiac surgery, low cardiac output syndrome, machine learning, predictive model, risk stratification

## Abstract

**Background:**

This study aimed to develop machine learning models to predict Low Cardiac Output Syndrome (LCOS) in patients following cardiac surgery using machine learning algorithms.

**Methods:**

The clinical data of cardiac surgery patients in Nanjing First Hospital between June 2019 and November 2020 were retrospectively extracted from the electronic medical records. Six conventional machine learning algorithms, including logistic regression, support vector machine, decision tree, random forest, extreme gradient boosting and light gradient boosting machine, were employed to construct the LCOS predictive models with all predictive features (full models) and selected predictive features (reduced models). The discrimination of these models was evaluated by the area under the receiver operating characteristic curve (AUC) and the calibration of the models was assessed by the calibration curve. Shapley Additive explanation (SHAP) and Local Interpretable Model-Agnostic Explanations (LIME) were used to interpret the predictive models.

**Results:**

Data from 1,585 patients [982 (62.0%) were male, aged 18 to 88, 212 (13.4%) with LCOS] were employed to train and validate the LCOS models. Among the full models, the RF model (AUC: 0.909, 95% CI: 0.875–0.943; Sensitivity: 0.849, 95% CI: 0.724–0.933; Specificity: 0.835, 95% CI: 0.796–0.869) and the XGB model (AUC: 0.897, 95% CI: 0.859–0.935; Sensitivity: 0.830, 95% CI: 0.702–0.919; Specificity: 0.809, 95% CI: 0.768–0.845) exhibited well predictive power for LCOS. Eleven predictive features including left ventricular ejection fraction (LVEF), first post-operative blood lactate (Lac), left ventricular diastolic diameter (LVDd), cumulative time of mean artery blood pressure (MABP) lower than 65 mmHg (MABP < 65 time), hypertension history, platelets level (PLT), age, blood creatinine (Cr), total area under curve above threshold central venous pressure (CVP) 12 mmHg and 16 mmHg, and blood loss during operation were used to build the reduced models. Among the reduced models, RF model (AUC: 0.895, 95% CI: 0.857–0.933; Sensitivity: 0.830, 95% CI: 0.702–0.919; Specificity: 0.806, 95% CI: 0.765–0.843) revealed the best performance. SHAP and LIME plot showed that LVEF, Lac, LVDd and MABP < 65 time significantly contributed to the prediction model.

**Conclusion:**

In this study, we successfully developed several machine learning models to predict LCOS after surgery, which may avail to risk stratification, early detection and management of LCOS after cardiac surgery.

## Introduction

Low Cardiac Output Syndrome (LCOS), a clinical manifestation of insufficient cardiac output and peripheral tissue perfusion, was first proposed by Rao et al. (1). Previous studies have shown that all-cause mortality in LCOS ranges from 14.8 to 62.5% in the short term (1 month post onset) and 21.4–36.6% in the long term (2 months to 1 year post onset) (2). LCOS following cardiac surgery not only leads to tissue malperfusion, but also multiple organ dysfunction of brain, lung, liver, kidney, and gastrointestinal tract, thereby increasing health care resource utilization and associated costs (3). More importantly, LCOS may be a state of reversible cardiac output (CO) reduction after cardiac surgery and early recognition and appropriate treatment of LCOS may avoid its progression to refractory cardiogenic shock and improve clinical outcomes, with early detection being of great significance (4–9).

The most common definition of LCOS (1) includes a decrease in the cardiac output index (CI) to < 2.2 L/min/m^2^ and a systolic blood pressure of < 90 mmHg, in conjunction with signs of tissue malperfusion (cold periphery, clammy skin, confusion, oliguria, elevated lactate level) in the absence of hypovolemia. Accordingly, it is of necessity to monitor CO by the pulmonary artery catheter (PAC) or pulse indicator continuous cardiac output (PICCO). All these current monitoring technologies, however, are too costly to be routinely applied in the setting of patients undergoing cardiac surgery, which consequently increases the difficulty of early recognition and prevention. Studies showed that significant independent risk factors for LCOS include age, preoperative left ventricular ejection fraction (LVEF), emergency surgery, temperature during cardiopulmonary bypass (CPB), application of cardioprotective drugs and echocardiographic parameters (10–12). Nevertheless, there were few studies on prediction models for LCOS. Therefore, this study was aimed to apply machine learning to develop models for the precise prediction of LCOS following cardiac surgery using preoperative variables and intraoperative time-series data, with the potential to avail early recognition and management of LCOS.

## Materials and methods

### Data sources and study population

This retrospective study was conducted on 1,681 consecutive patients admitted and received cardiac surgery at Nanjing First Hospital from June 2019 to November 2020. Patients who received cardiac surgery during the study period were recruited as the study objects, including but not limited to coronary artery bypass, heart valve surgery, aortic dissection (AD) repair surgery, etc. Exclusion criteria: (1) Patients under 18 years of age. (2) Patients who died or were discharged during or within 48 h after the operation. (3) Patients with incomplete clinical data, such as pre-operation echocardiographic measurements or intraoperative hemodynamic data. Data were collected from electronic medical records (EMR) database, and approval was gained from the Ethics Committee of Nanjing First Hospital (KY20220518-KS-01).

### Definition of low cardiac output syndrome

According to previous reports (10, 13), the criteria for LCOS in our study included: (1) Patients with a cardiac index (CI) reduced to < 2.2 L/min/m^2^; (2) Patients with systolic blood pressure < 90 mmHg, in conjunction with signs of tissue hypoperfusion [oliguria (urine output < 1 ml/kg.h), elevated lactate level > 3.0 mmol/L]; (3) Patients requiring mechanical circulatory support or inotropic agents (dopamine or dobutamine at least 4 μg/kg.min for a minimum of 12 h and/or epinephrine at least 0.2 μg/kg.min and/or milrinone at least 0.02 μg/kg.min and/or levosimendan at least 0.05 μg/kg.min) to maintain hemodynamics after optimizing preload. Patients who received vasoconstricting medication to increase peripheral vascular resistance in the presence of normal cardiac output were not considered to have LCOS.

### Data collection and preprocessing of data

Clinical variables extracted from electronic medical records (EMR) database included demographics: age, sex, height, weight; comorbidities: hypertension, diabetes, myocardial infarction, hyperlipidemia, cerebral vascular disease, atrial fibrillation, chronic obstructive pulmonary disease (COPD), congestive heart failure, renal disease, liver disease; preoperative echocardiographic parameters: left ventricular diastolic diameter (LVDd), left atrial diameter (LAD), interventricular septum thickness in diastole (IVSd), left ventricular posterior wall thickness (LVPWT), pulmonary artery systolic pressure (PASP), left ventricular ejection fraction (LVEF); preoperative laboratory results: white blood cell count (WBC), neutrophil ratio (NEU), lymphocyte ratio (LYM), platelets level (PLT), hemoglobin (Hb), blood creatinine (Cr), blood urea nitrogen (BUN), aspartate aminotransferase (AST), alanine aminotransferase (ALT), total protein (TP), total bilirubin (TB), low density lipoprotein (LDL), creatine kinase-MB (CKMB), triiodothyronine (T3), thyroxine (T4), thyroid stimulating hormone (TSH), brain natriuretic peptide (BNP); operation information: operation time, cardiopulmonary bypass (CPB) time, aortic occlusion (AO) time, Emergency surgery, urine output (UO) during operation, blood loss during operation, operation type; intraoperative hemodynamics: mean arterial blood pressure (MABP), central venous pressure (CVP); postoperative hemodynamics: cardiac output (via pulmonary artery catheter for some patients), systolic artery blood pressure (SABP), CVP, inotropes (dopamine, dobutamine, epinephrine, milrinone, and levosimendan) usage, urine output and first post-operative blood lactate levels (within 30 min post operation), prognosis variables: mechanical ventilation (MV) time, ICU stay time and hospital stay time. Renal disease was defined as preoperative glomerular filtration rate < 30 ml/min/1.73 m^2^ (body surface area) (14). Hyperlipidemia was defined as total cholesterol > 200 mg/dl and/or triglyceridemic value > 150 mg/dl. Other comorbidities were identified from diagnosis before operation using the International Classification of Disease, Tenth edition (ICD-10). ICD-10 codes used for the identification of comorbidities are outlined in Supporting Information (Supplementary Table 1).

During operation, MABP and CVP were continuously monitored using invasive peripheral artery, central vein or pulmonary artery catheter and saved as time-series data. Artifactual data were removed according to previously published criteria (15). Thresholds for MABP (< 65, < 60, < 55, < 50 mmHg) and CVP (> 12, > 16, > 20 mmHg) were used to assess the site of hypotension and central venous congestion occurred during operation. To comprehensively assess the time-series data, cumulative time under or above thresholds, total area under curve under or above threshold (AUT) and time weighted average (TWA) of MABP and CVP for corresponding threshold were calculated based on a previous study (16).

### Model construction and evaluating

The entire dataset was randomly stratified into the training and test sets (7:3), meaning that the ratio of patients with LCOS to those without LCOS was maintained consistent in both subsets. The training set was applied to train the model with 10-fold cross-validation and test set was used later to assess the models’ performance. All variables with a missing rate > = 10% were excluded from the analysis (Supplementary Figure 1). Variables with a missing rate < 10% were imputed by the k-nearest neighbors (KNN) imputation procedure (17). The low incidence of LCOS and the large number of variables we included in this study made it typical unbalanced high dimension data, Synthetic Minority Oversampling Technique (SMOTE) was applied to overcome this imbalance.

Six conventional machine learning algorithms were employed to construct the LCOS prediction models will all variables (full models), including logistic regression (LR), support vector machine (SVM), decision tree (DT), random forest (RF), extreme gradient boosting (XGB), and light gradient boosting machine (LGB).

Boruta and the least absolute shrinkage and selection operator (LASSO) were used to select the optimal subset of variables. All variables confirmed as important by the Boruta algorithm were entered the LASSO regressing. Finally, variables identified by LASSO regression were included for constructing reduced models using the same six machine learning algorithms.

### Statistical analyses

Baseline characteristics of patients in the training and test sets were compared. Measurements conforming to a normal distribution were described as mean ± standard deviation. Student’s *t*-test was employed for comparisons. Measurement data that did not conform to a normal distribution were denoted as median [lower quartile-upper quartile]. Wilcoxon rank-sum tests were performed to draw comparisons. The enumeration data were represented as frequency and percentage and compared by performing Pearson χ^2^ test. Fisher’s exact test was performed under the expected frequencies of one or more cells less than 5. The difference was considered with statistical significance at *P* < 0.05.

The discriminations of models were evaluated by the area under curve (AUC) of the receiver operating characteristic (ROC), accuracy, sensitivity, specificity and calibration of the models were assessed by the calibration curve and Brier score. Shapley Additive explanation (SHAP) and Local Interpretable Model-Agnostic Explanations (LIME) were used to provide consistent and locally accurate values for each variable within the prediction models. All analyses were conducted in R (version 3.6.3) and Python (version 3.7).

## Results

Overall, the eligibility of 1,681 patients who underwent cardiac surgery and were admitted to the Cardiovascular ICU of Nanjing First Hospital, Nanjing Medical University, from June 2019 to November 2020 was assessed. The excluded cases were as follows: 35 cases were younger than 18 years old, 8 patients were dead or discharged within 48 h after surgery and 53 patients had uncompleted data. Finally, 1585 patients [982 (62.0%) male, 18 to 88 years old] were enrolled for analyses. Among them, 386 (24.4%) patients received PAC insertion during the surgery, and the proportion of PAC use varied by surgery types (Supplementary Table 2). Among patients with PAC, 61 (15.8%) were diagnosed with LCOS by CI criterion, and among the other 1,199 patients without PAC, 151 (12.6%) were diagnosed with LCOS by other criteria. Overall, 212 (13.4%) patients developed LCOS postoperatively. Compared to patients without LCOS, patients with LCOS had prolonged MV time (20.25 [13.08,40.38] vs. 9.50 [7.00,15.33] hours, *P* < 0.001), longer ICU stay time (3.0 [2.0,6.0] vs. 1.0 [1.0,2.0] days, *P* < 0.001) and hospital stay time (21.0 [16.0,27.0] vs. 17.0 [14.0,21.0] days, *P* < 0.001). There was no significant difference in morbidity between patients with LCOS diagnosed by CI criterion and other criteria (15.6 vs. 12.5%, *P* = 0.127), and patients with LCOS diagnosed by different criteria had similar prognoses (Supplementary Figure 2), indicating consistency between the different criteria.

We randomized 70% of these 1,585 patients into the training set and the remaining 30% into the test set. The clinical variables of patients in training and test set are listed in Table 1. There was no significant difference between patients in training and test sets for these variables.

**TABLE 1:** Patient characteristics and clinical variables.

	Training set (*N* = 1,109)	Test set (*N* = 476)	*P*-value
**Demographic data**			
Age (years)	61.26 ± 12.02	60.98 ± 11.47	0.658
Male, n (%)	693 (62.5%)	289 (60.7%)	0.541
Height (cm)	165.38 ± 8.39	165.33 ± 8.64	0.920
Weight (kg)	65.83 ± 11.93	65.94 ± 11.68	0.860
**Comorbidities**			
Hypertension, n (%)	568 (51.2%)	227 (47.7%)	0.218
Diabetes, n (%)	256 (23.1%)	93 (19.5%)	0.135
Myocardial infarction, n (%)	62 (5.6%)	26 (5.5%)	1
Hyperlipidemia, n (%)	233 (21.0%)	87 (18.3%)	0.240
Cerebral vascular disease, n (%)	105 (9.5%)	33 (6.9%)	0.123
Atrial Fibrillation, n (%)	244 (22.0%)	100 (21.0%)	0.709
COPD, n (%)	39 (3.5%)	19 (4.0%)	0.752
Heart failure, n (%)	445 (40.1%)	206 (43.3%)	0.266
Kidney disease, n (%)	78 (7.0%)	29 (6.1%)	0.565
Liver disease, n (%)	39 (3.5%)	11 (2.3%)	0.270
**Preoperative ECHO**			
LVDd (mm)	53.98 ± 9.86	53.78 ± 9.09	0.695
LVPWT (mm)	9.85 ± 2.21	9.74 ± 1.40	0.218
LVEF (%)	59.11 ± 9.08	59.05 ± 8.42	0.906
**Laboratory**			
WBC (10^9^/L)	6.76 ± 2.81	6.64 ± 2.45	0.393
NEU (%)	62.84 ± 10.71	62.13 ± 10.62	0.224
LYM (%)	25.42 ± 9.20	27.13 ± 9.52	0.170
PLT (10^9^/L)	188.90 ± 66.07	183.33 ± 63.05	0.113
Hb (g/L)	131.07 ± 18.69	131.92 ± 19.13	0.413
Cr (mmol/L)	83.83 ± 77.54	80.54 ± 59.35	0.359
BUN (mmol/L)	6.67 ± 2.94	6.66 ± 3.17	0.981
AST (U/L)	22.00 [17.00, 30.00]	22.00 [17.00, 30.00]	0.810
Lac (mmol/L)	2.23 ± 1.80	2.26 ± 1.87	0.745
**Operative variables**			
Operation time (hour)	4.39 ± 1.37	4.38 ± 1.36	0.903
CPB time (min)	101.62 ± 66.24	100.45 ± 54.72	0.714
AO time (min)	69.85 ± 54.37	67.61 ± 38.54	0.351
Emergency surgery, n (%)	117 (10.6%)	41 (8.6%)	0.276
Urine output (ml/kg/h)	3.18 ± 1.99	3.16 ± 2.01	0.869
Blood loss (ml)	1165.90 ± 699.38	1155.65 ± 612.52	0.770
**Operation type**			
CABG only, n (%)	311 (28.0%)	130 (27.3%)	0.813
Valve surgery only, n (%)	420 (37.9%)	188 (39.5%)	0.58
CABG + valve surgery, n (%)	100 (9.0%)	53 (11.1%)	0.224
Congenital surgery, n (%)	70 (6.3%)	24 (5.0%)	0.387
Heart transplant, n (%)	14 (1.3%)	4 (0.8%)	0.640
Aortic dissection repair, n (%)	84 (7.6%)	23 (4.8%)	0.059
Other surgery, n (%)	110 (10.3%)	54 (11%)	0.437
**Hemodynamic data**			
MABP < 65 time (min)	129.87 ± 77.61	128.13 ± 72.32	0.667
MABP < 60 time (min)	94.73 ± 66.78	92.88 ± 60.96	0.592
MABP < 55 time (min)	65.72 ± 53.95	63.61 ± 48.11	0.441
MABP < 50 time (min)	41.72 ± 41.44	40.34 ± 35.95	0.503
MABP_AUT_65 (mmHg*min)	1583.54 ± 1235.09	1535.92 ± 1087.10	0.443
MABP_AUT_60 (mmHg*min)	1005.51 ± 899.36	966.54 ± 780.09	0.385
MABP_AUT_55 (mmHg*min)	592.29 ± 617.00	563.21 ± 524.40	0.338
MABP_AUT_50 (mmHg*min)	313.91 ± 393.54	292.96 ± 323.69	0.270
MABP_TWA_65 (mmHg)	11.30 ± 3.87	11.31 ± 3.72	0.938
MABP_TWA_60 (mmHg)	9.54 ± 3.78	9.54 ± 3.42	0.982
MABP_TWA_55 (mmHg)	7.79 ± 3.62	7.80 ± 3.41	0.969
MABP_TWA_50 (mmHg)	6.07 ± 3.39	6.07 ± 3.25	0.995
CVP > 12 time (min)	15.00 [0.00, 65.00]	20.00 [5.00, 60.00]	0.815
CVP > 16 time (min)	0.00 [0.00, 10.00]	0.00 [0.00, 10.00]	0.447
CVP > 20 time (min)	0.00 [0.00, 0.00]	0.00 [0.00, 5.00]	0.129
CVP_AUT_12 (mmHg*min)	50.00 [0.00, 225.00]	67.50 [5.00, 220.00]	0.373
CVP_AUT_16 (mmHg*min)	0.00 [0.00, 50.00]	0.00 [0.00, 60.00]	0.231
CVP_AUT_20 (mmHg*min)	0.00 [0.00, 0.00]	0.00 [0.00, 10.00]	0.081
CVP_TWA_12 (mmHg)	2.22 [0.00, 4.00]	2.27 [1.00, 4.57]	0.122
CVP_TWA _16 (mmHg)	0.00 [0.00, 2.86]	0.00 [0.00, 4.00]	0.081
CVP_TWA _20 (mmHg)	0.00 [0.00, 0.00]	0.00 [0.00, 2.00]	0.053
**LCOS**			
Yes, n (%)	148 (13.3%)	64 (13.4%)	1

ECHO, echocardiography; LVDd, left ventricular diastolic diameter; LVPWT, left ventricular posterior wall thickness; LVEF, left ventricular ejection fraction; WBC, white blood cell count; NEU, neutrophil properties; Cr, blood creatinine; BUN, blood urea nitrogen; AST, aspartate aminotransferase; Lac, blood lactate; CPB, cardiopulmonary bypass; AO, aortic occlusion; CABG, coronary artery bypass graft; MABP < 65 time, cumulative time of mean artery blood pressure lower than 65 mmHg; MABP < 60 time, cumulative time of mean artery blood pressure lower than 60 mmHg; MABP < 55 time, cumulative time of mean artery blood pressure lower than 55 mmHg; MABP < 50 time, cumulative time of mean artery blood pressure lower than 50 mmHg; MABP_AUT_65, total area under curve below threshold mean artery blood pressure 65 mmHg; MABP_AUT_60, total area under curve below threshold mean artery blood pressure 60 mmHg; MABP_AUT_55, total area under curve below threshold mean artery blood pressure 55 mmHg; MABP_AUT_50, total area under curve below threshold mean artery blood pressure 50 mmHg; MABP_TWA_65, time weighted average mean artery blood pressure below threshold 65 mmHg; MABP_TWA_60, time weighted average mean artery blood pressure below threshold 60 mmHg; MABP_TWA_55, time weighted average mean artery blood pressure below threshold 55 mmHg; MABP_TWA_50, time weighted average mean artery blood pressure below threshold 50 mmHg; CVP > 12 time, cumulative time of central venous pressure upper than 12 mmHg; CVP > 16 time, cumulative time of central venous pressure upper than 16 mmHg; CVP > 20 time, cumulative time of central venous pressure upper than 20 mmHg; CVP_AUT_12, total area under curve above threshold central venous pressure 12 mmHg; CVP_AUT_16, total area under curve above threshold central venous pressure 16 mmHg; CVP_AUT_20, total area under curve above threshold central venous pressure 20 mmHg; CVP_TWA_12, time weighted average central venous pressure above threshold 12 mmHg; CVP_TWA_16, time weighted average central venous pressure above threshold 16 mmHg; CVP_TWA_20, time weighted average central venous pressure above threshold 20 mmHg; LCOS, low cardiac output syndrome.

**FIGURE 1:**
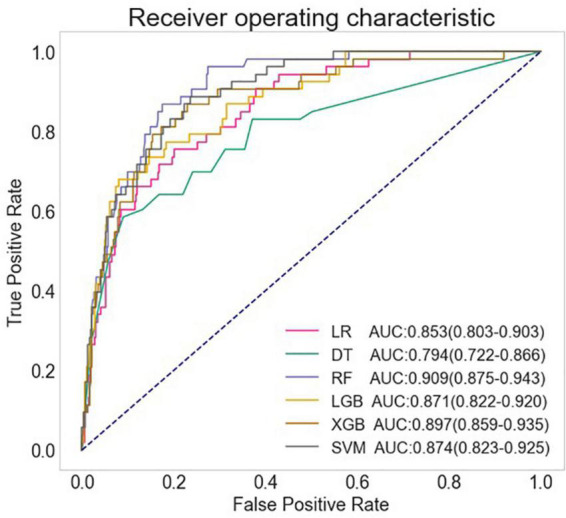
Comparison of AUCs among different machine learning models. LR, logistic regression; DT, decision tree; RF, random forest classifier; LGB, light gradient boosting machine; XGB, extreme gradient boosting machine; SVM, support vector machine.

**FIGURE 2:**
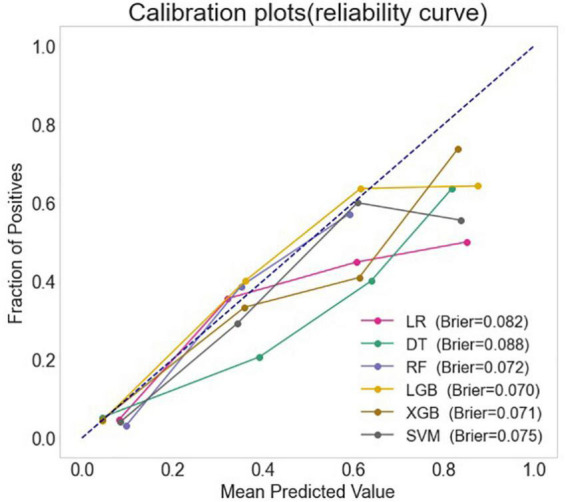
The calibration curves and the Brier score of different machine learning models. LR, logistic regression; DT, decision tree; RF, random forest classifier; LGB, light gradient boosting machine; XGB, extreme gradient boosting machine; SVM, support vector machine.

The full models were conducted with all variables, using the six algorithms including LR, DT, SVM, RF, XGB, and LGB for LCOS predicting, and the AUC, accuracy, sensitivity, and specificity of each full model on test set were presented in Figure 1 and Table 2. Among the full models, the RF model (AUC: 0.909, 95% CI: 0.875–0.943; Sensitivity: 0.849, 95% CI: 0.724–0.933; Specificity: 0.835, 95% CI: 0.796–0.869) and the XGB model (AUC: 0.897, 95% CI: 0.859–0.935; Sensitivity: 0.830, 95% CI: 0.702–0.919; Specificity: 0.809, 95% CI: 0.768–0.845) showed well predictive power for LCOS. The main parameters of the full RF model were set as follows: bootstrap = True, criterion = “gini,” n_estimators = 500, max_depth = None, min_samples_leaf = 1, min_sample_split = 2. The main parameters of the full XBG model were set as follows: n_estimators = 200, learning_rate = 0.1, max_depth = 9, gamma = 0. The calibration plot and Brier score indicated all the full models have well calibration (Figure 2).

**TABLE 2:** The performance of each model.

	Model	AUC	Accuracy	Sensitivity	Specificity
Full models	LR	0.853 (0.803–0.903)	0.761 (0.72–0.798)	0.755 (0.617–0.862)	0.761 (0.718–0.801)
	DT	0.794 (0.722–0.866)	0.716 (0.674–0.756)	0.698 (0.557–0.817)	0.719 (0.673–0.761)
	RF	0.909 (0.875–0.943)	0.836 (0.800–0.868)	0.849 (0.724–0.933)	0.835 (0.796–0.869)
	LGB	0.871 (0.822–0.920)	0.813 (0.775–0.847)	0.755 (0.617–0.862)	0.820 (0.780–0.856)
	XGB	0.897 (0.859–0.935)	0.811 (0.773–0.845)	0.830 (0.702–0.919)	0.809 (0.768–0.845)
	SVM	0.874 (0.823–0.925)	0.813 (0.775–0.847)	0.774 (0.638–0.877)	0.818 (0.778–0.854)
Reduced models	LR	0.815 (0.754–0.877)	0.708 (0.665–0.748)	0.717 (0.577–0.832)	0.707 (0.661–0.75)
	DT	0.740 (0.661–0.818)	0.580 (0.534–0.625)	0.774 (0.638–0.877)	0.556 (0.507–0.604)
	RF	0.895 (0.857–0.933)	0.809 (0.771–0.843)	0.830 (0.702–0.919)	0.806 (0.765–0.843)
	LGB	0.853 (0.800–0.906)	0.767 (0.726–0.804)	0.792 (0.659–0.892)	0.764 (0.72–0.803)
	XGB	0.854 (0.803–0.905)	0.752 (0.711–0.790)	0.792 (0.659–0.892)	0.747 (0.703–0.788)
	SVM	0.853 (0.800–0.905)	0.775 (0.735–0.812)	0.774 (0.638–0.877)	0.775 (0.733–0.814)

AUC, area under curve of receiver operating characteristic; LR, logistic regression; DT, decision tree; RF, random forest classifier; LGB, light gradient boosting machine; XGB, extreme gradient boosting machine; SVM, support vector machine.

Feature selection was performed by the following two steps. First, Boruta algorithm was employed and 35 features were confirmed important to the prediction of LCOS (Supplementary Figure 3). Then, Lasso regression was applied to select the best subset features from the 35 confirmed important features (Supplementary Figure 4). Eleven variables were finally selected by Boruta and LASSO features selection procedure, including LVEF, lactate (Lac), LVDd, cumulative time of mean artery blood pressure (MABP) lower than 65 mmHg (MABP < 65 time), hypertension history, PLT, age, blood Cr, AST, total area under curve above CVP 12 mmHg (CVP_AUT_12), total area under curve above threshold CVP 16 mmHg (CVP_AUT_16) and blood loss during operation. Six reduced models with these eleven variables and the same six algorithms were then developed. Among the reduced models, among which, RF model (AUC:0.895, 95% CI: 0.857–0.933; Sensitivity:0.830, 95% CI: 0.702–0.919; Specificity: 0.806, 95% CI: 0.765–0.843) revealed the best performance. The main parameters of the reduced RF model were set as follows: bootstrap = True, criterion = “gini,” n_estimators = 700, max_depth = None, min_samples_leaf = 1, min_sample_split = 2. The AUC, accuracy, sensitivity and specificity of the full and reduced models are presented in Table 2.

**FIGURE 3:**
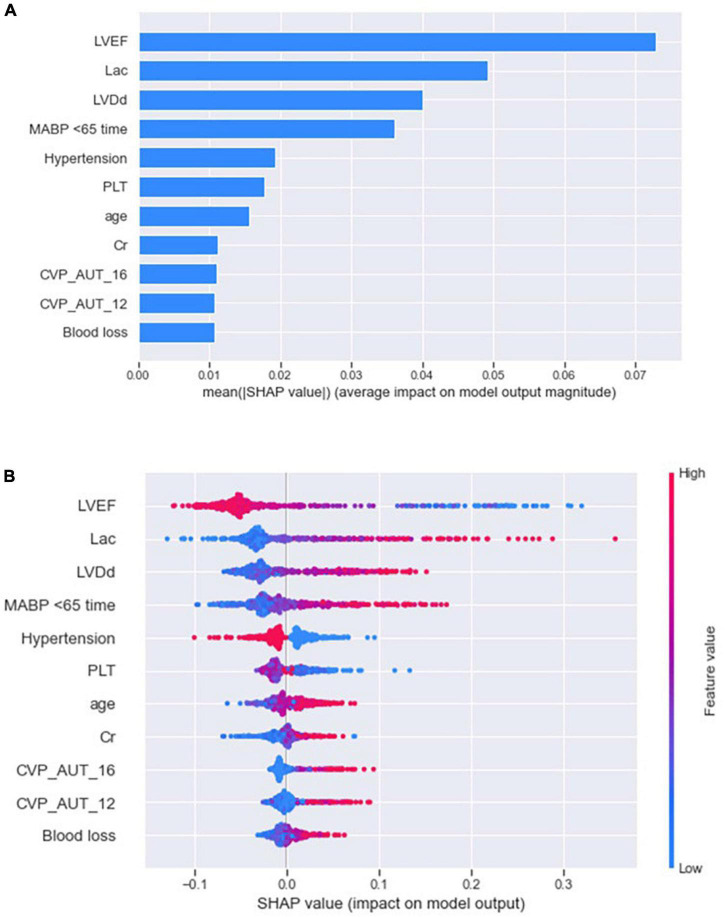
SHAP summary plot of the reduced RF model. The plot showed the importance of each variable **(A)** and the specific distribution between variables and Shapely value **(B)** using SHAP algorithm.

**FIGURE 4:**
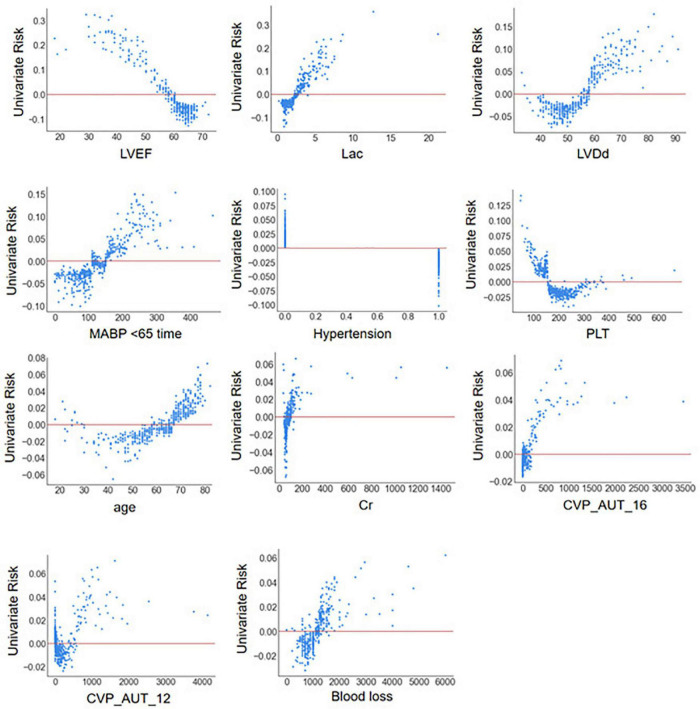
SHAP dependence plot of the reduced RF model. ECHO, echocardiography; LVDd, left ventricular diastolic diameter; LVPWT, left ventricular posterior wall thickness; LVEF, left ventricular ejection fraction; WBC, white blood cell count; NEU, neutrophil properties; Cr, blood creatinine; BUN, blood urea nitrogen; AST, aspartate aminotransferase; Lac, blood lactate; CPB, cardiopulmonary bypass; AO, aortic occlusion; CABG, coronary artery bypass graft; MABP < 65 time, cumulative time of mean artery blood pressure lower than 65 mmHg; MABP < 60 time, cumulative time of mean artery blood pressure lower than 60 mmHg; MABP < 55 time, cumulative time of mean artery blood pressure lower than 55 mmHg; MABP < 50 time, cumulative time of mean artery blood pressure lower than 50 mmHg; MABP_AUT_65, total area under curve below threshold mean artery blood pressure 65 mmHg; MABP_AUT_60, total area under curve below threshold mean artery blood pressure 60 mmHg; MABP_AUT_55, total area under curve below threshold mean artery blood pressure 55 mmHg; MABP_AUT_50, total area under curve below threshold mean artery blood pressure 50 mmHg; MABP_TWA_65, time weighted average mean artery blood pressure below threshold 65 mmHg; MABP_TWA_60, time weighted average mean artery blood pressure below threshold 60 mmHg; MABP_TWA_55, time weighted average mean artery blood pressure below threshold 55 mmHg; MABP_TWA_50, time weighted average mean artery blood pressure below threshold 50 mmHg; CVP > 12 time, cumulative time of central venous pressure upper than 12 mmHg; CVP > 16 time, cumulative time of central venous pressure upper than 16 mmHg; CVP > 20 time, cumulative time of central venous pressure upper than 20 mmHg; CVP_AUT_12, total area under curve above threshold central venous pressure 12 mmHg; CVP_AUT_16, total area under curve above threshold central venous pressure 16 mmHg; CVP_AUT_20, total area under curve above threshold central venous pressure 20 mmHg; CVP_TWA_12, time weighted average central venous pressure above threshold 12 mmHg; CVP_TWA_16, time weighted average central venous pressure above threshold 16 mmHg; CVP_TWA_20, time weighted average central venous pressure above threshold 20 mmHg; LCOS, low cardiac output syndrome.

**FIGURE 5:**
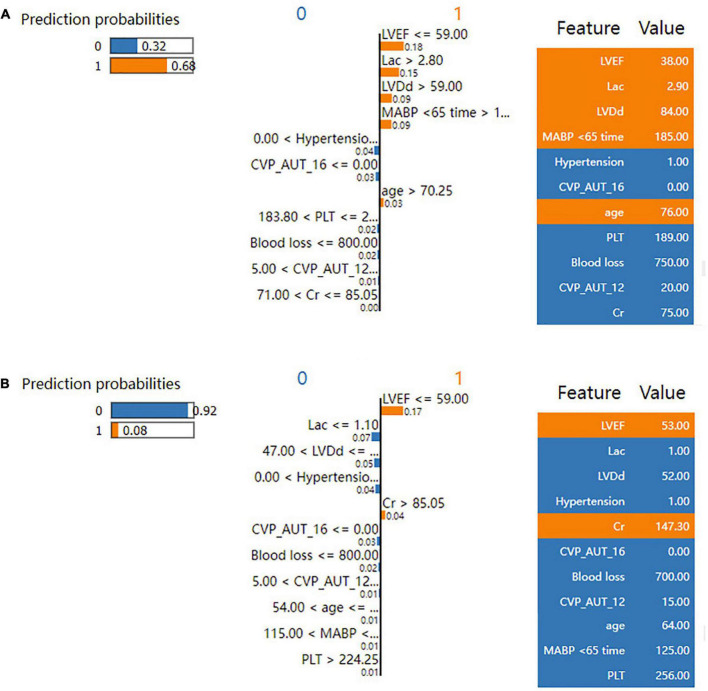
LIME plot for individual case explanation on two random patients for the test set of the reduced RF model. LIME plot included one patient with LCOS **(A)** and one patient without LCOS **(B)**, explained by LIME algorithm. ECHO, echocardiography; LVDd, left ventricular diastolic diameter; LVPWT, left ventricular posterior wall thickness; LVEF, left ventricular ejection fraction; WBC, white blood cell count; NEU, neutrophil properties; Cr, blood creatinine; BUN, blood urea nitrogen; AST, aspartate aminotransferase; Lac, blood lactate; CPB, cardiopulmonary bypass; AO, aortic occlusion; CABG, coronary artery bypass graft; MABP < 65 time, cumulative time of mean artery blood pressure lower than 65 mmHg; MABP < 60 time, cumulative time of mean artery blood pressure lower than 60 mmHg; MABP < 55 time, cumulative time of mean artery blood pressure lower than 55 mmHg; MABP < 50 time, cumulative time of mean artery blood pressure lower than 50 mmHg; MABP_AUT_65, total area under curve below threshold mean artery blood pressure 65 mmHg; MABP_AUT_60, total area under curve below threshold mean artery blood pressure 60 mmHg; MABP_AUT_55, total area under curve below threshold mean artery blood pressure 55 mmHg; MABP_AUT_50, total area under curve below threshold mean artery blood pressure 50 mmHg; MABP_TWA_65, time weighted average mean artery blood pressure below threshold 65 mmHg; MABP_TWA_60, time weighted average mean artery blood pressure below threshold 60 mmHg; MABP_TWA_55, time weighted average mean artery blood pressure below threshold 55 mmHg; MABP_TWA_50, time weighted average mean artery blood pressure below threshold 50 mmHg; CVP > 12 time, cumulative time of central venous pressure upper than 12 mmHg; CVP > 16 time, cumulative time of central venous pressure upper than 16 mmHg; CVP > 20 time, cumulative time of central venous pressure upper than 20 mmHg; CVP_AUT_12, total area under curve above threshold central venous pressure 12 mmHg; CVP_AUT_16, total area under curve above threshold central venous pressure 16 mmHg; CVP_AUT_20, total area under curve above threshold central venous pressure 20 mmHg; CVP_TWA_12, time weighted average central venous pressure above threshold 12 mmHg; CVP_TWA_16, time weighted average central venous pressure above threshold 16 mmHg; CVP_TWA_20, time weighted average central venous pressure above threshold 20 mmHg; LCOS, low cardiac output syndrome.

The SHAP summary plot (Figure 3) and dependence plot (Figure 4) represented the contributions of these eleven variables to the prediction of the RF model, with SHAP values above zero indicating an increased risk of developing LCOS and SHAP values below zero indicating a decreased risk of LCOS. For example, SHAP values for high LVEF (red) were usually less than zero, indicating a decreased risk of LCOS in patients with higher LVEF. In addition, Figure 3A displays the ranking of the features based on the average absolute SHAP value. Among the eleven variables, LVEF, Lac, LVDd and MABP < 65 time were the four variables with the greatest influence on prediction power. Lower LVEF, higher Lac, larger LVDd and longer MABP < 65 time indicated an increased possibility of the onset of LCOS. We randomly selected two patients with LCOS (Figure 5A) and without LCOS (Figure 5B) and used LIME algorithm to interpret how they were predicted to be have a 68% possibility of LCOS and 92% possibility without LCOS. The first patient (Figure 5A) was predicted to be with possibility of prospective LCOS due to low LVEF (38%), high Lac (2.9 mmol/L), large LVDd (84 mm), long MABP < 65 time (185 min) and advanced age (76 years). The second patient (Figure 5B) was predicted to be without prospective LCOS due to relatively normal variables: Lac (1.0 mmol/L), LVDd (52 mm), hypertension, CVP_AUT_16 (0 min), Blood loss (700 ml), CVP_AUT_12 (15 min), age (64 years), MAPB < 65 time (125 min), PLT (256 * 10^9/L).

## Discussion

Big data and machine learning are enabling the shift from conventional to customized treatment, which could soon result in the birth of a new health system (18, 19). To the best of our knowledge, no machine learning prediction model has been established to predict the occurrence of LCOS following cardiac surgery. In the present study, in the cooperation of clinicians and information technology engineers, we successfully developed several machine learning models to predict LCOS following cardiac surgery. Six conventional machine learning algorithms were employed to construct the LCOS prediction models, including LR, SVM, DT, RF, XGB, and LGB, indicating that RF and XGB models exhibited the best performance. RF is a homologous ensemble algorithm that constructs a great number of decision trees during training, which helps to build robust prediction models with able to deal with non-linear data. XGB is a distributed algorithm with fast operation speed and high fault tolerance, which could accurately predicts the outcome of multiple diseases in ICU (20–22). Commendably, our study demonstrated that the performance of machine learning models was significantly superior to the traditional logistic regression models in the prediction of LCOS following cardiac surgery.

We adopted a dual definition of LCOS, similar to the prior studies (10, 13), with the CI criterion requiring perioperative PAC monitoring. However, even in the field of cardiothoracic surgery, the usage of the PAC has declined over the years. The most accurate way to evaluate the pulmonary artery and cardiac output in patients with pulmonary hypertension and heart failure, however, is through the use of PAC. The large proportion of PAC usage in various procedures, such as heart transplant, adult congenital surgery, and challenging combination CABG + valve surgery, can be attributed to its potential benefit in patients with a high risk of RV failure (8, 23) (Supplementary Table 2). PAC could continually provide important hemodynamic measurements like pulmonary circulation resistance, right heart afterload, cardiac output, etc. Those measurements are imperative in perioperative management of critically ill patients after those types of surgery. However, PAC use was reported to be associated with a poorer outcome in patients receiving cardiac surgical. As an invasive hemodynamic monitoring method, the difficulty of placement and consequent side effects may contribute to iatrogenic adverse outcomes for patients (24). The similar prognosis outcome between LCOS patients diagnosed by CI criterion and other criteria indicated consistency across different criteria. Importantly, our prediction models could provide a non-invasive, precious, interpretable way to predict LCOS, perhaps reducing the need for intrusive monitoring techniques like PAC.

Low Cardiac Output Syndrome could be corrected by timely and effective intervention and a variety of therapeutic strategies can be applied to the treatment of LCOS, when it is early recognized, including optimization of ventricular preload and afterload; inotropic agents; positive pressure ventilation; heart rhythm and rate control; metabolic and hormonal disorders correction; and in extreme circumstances mechanical circulatory support (8, 9, 25, 26). Most features we included in the full and reduced models were preoperative clinical and intraoperative hemodynamic variables. Our prediction model could be integrated into the EMR system for use in everyday practice, and the HER database could automatically provide the model with the data it needs. In the very early phase after surgery, LCOS models could provide LCOS risk prediction and shed a light on further strategies for postoperative management and initiation of individualized therapy.

The present study showed that LVEF, Lac, LVDd, MABP < 65 time, hypertension, PLT, age, Cr, CVP_AUT_16, CVP_AUT_12, and blood loss significantly contributed to the prediction. The reduced RF model using these features also showed little discrimination loss in the prediction of LCOS (AUC:0.895 vs. 0.909) but it could significantly increase the efficiency and convenience, which may contribute to risk stratification and short-term decision making for LCOS.

Traditionally, machine learning models have been less interpretable when compared to traditional regression models. This black-box behavior has hindered their application in clinical settings. To enhance the interpretability of machine learning, we utilized SHAP and LIME interpreter techniques to visualize how features affect the prediction of LCOS, both globally and individually accordingly. SHAP summary plot revealed that LVEF, Lac, LVDd and MABP < 65 time were the most significant predictors of LCOS, with lower LVEF, higher Lac, larger LVDd and longer MABP < 65 time indicating increased possibility of the prospective onset of LCOS. LVEF is the most widely used estimate of left ventricular systolic function and a decreased LVEF is an independent risk factor for LCOS (27, 28). Serum lac is a well-recognized biomarker of tissue perfusion, and elevated lac can serve as a sensitive indicator of LCOS. Ventricular dilatation is a common compensatory response to decreased myocardial contractility (29), which can explain the association between enlarged LVDd and the risk of LCOS. MABP < 65 time also serves as a surrogate for a hypotension state subsequent to reduced cardiac output. We tried multiple thresholds of MABP (< 65, < 60, < 55, < 50 mmHg) and MABP < 65 mmHg showed a better predictive value than other thresholds, suggesting 65 mmHg was a good MABP threshold regarding maintenance of tissue perfusion (30). In our study, patients with a history of hypertension were less likely to develop LCOS after cardiac surgery, which is consistent with a previous study (3). Hypertension is usually associated with myocardial hypertrophy and is accompanied by enhanced myocardial contractility. Notably, hypertension patients were also reported to have higher mortality after the onset of LCOS, because myocardial hypertrophy would exacerbate the deficiency of oxygen supply subsequent to LCOS (31).

Our study had several advantages when compared to previous studies. Firstly, our study included comprehensive variables including demographics, commodities, echocardiographic and laboratory measurements and operation related information including intraoperative hemodynamic data, which could reflect the patient’s profile in multiple dimensions. Secondly, we incorporated various hemodynamic time-series features that were considered difficult to incorporate in prediction models (32–34). In our previous study, we demonstrated an association between hemodynamic time-series data and postoperative organ dysfunction (35). It is well known that intraoperative hypotension and venous congestion may be a reflection of LCOS. We examined cumulative time, total area under curve and time weighted average under or above pre-specified thresholds other than using static measures as in other studies (36). In this way, we could assess both the duration and severity of hypotension and venous congestion, so as to better contribute to the prediction of LCOS.

Thirdly, we analyzed the predictive value of all features and selected features for LCOS prediction. Some studies made feature selection only based on linear regression or stepwise logistic regression, which may exclude features that were not statistically significant but have causal effects on the output variable due to non-linear relationships or interactions between the variables and outcomes (37). As a wrapper built around the random forest classification algorithm, Boruta performed classification by voting on multiple unbiased weak decision trees (38), which could deal with non-linear and complex relationships between the features and the outcome. Thus, our approach reduced the possibility of missing important or previously unreported features.

This study was subject to some limitations. First, we did not compare the performance of our models with previous LCOS risk scores because some of the variables required in the risk scores were not available. Second, the models have not been verified in the external validation queue. Third, this is a single center retrospective study. Further multi-center studies with external validation are needed to further verify our findings and prospective studies could be of more importance in assessing the performance of our predictive models.

## Conclusion

In the present study, we successfully developed several machine learning models to predict LCOS following cardiac surgery, which may avail to risk stratification, early detection and management of LCOS following cardiac surgery.

## Data availability statement

The original contributions presented in this study are included in the article/Supplementary material, further inquiries can be directed to the corresponding authors.

## Ethics statement

The study protocol was conducted in accordance with the Declaration of Helsinki and was approved by the Ethics Committee of Nanjing First Hospital, Nanjing Medical University (KY20220518-KS-01). Informed consent was not obtained due to the observational and anonymous nature of data collection.

## Author contributions

LH, HX, CG, DG, and CZ conceived the conception of the study. LH, HX, CG, HT, and XSh acquired the data. XSo, DG, and CZ participated in data analyses. CG and DG constructed the predictive model. LH, HX, CG, and HT prepared the first draft of the manuscript. CZ and DG led the project and supervised the study. All authors were involved in writing or editing the manuscript, read, and approved the final version of the manuscript.

## Supplementary material

Supplementary Figure 1 Missing value in the study.

Supplementary Figure 2 Prognosis variables in patients with LCOS diagnosed by CI criterion and other criteria and patients without LCOS, including mechanical ventilation time **(A)**, ICU stay time **(B)** and hospital stay time **(C)**.

Supplementary Figure 3 Feature importance determined through Boruta algorithm.

Supplementary Figure 4 Subset feature selection through Lasso regression.

## Abbreviations

LCOS: low cardiac output syndrome
EMR: electronic medical records
LR: logistic regression
SVM: support vector machine
DT: decision tree
RF: random forest
XGB: extreme gradient boosting
LGB: light gradient boosting machine
AUC: area under the receiver operating characteristic curve
SHAP: shapley additive explanation
LIME: local interpretable model-agnostic explanations
LVEF: left ventricular ejection fraction
CO: cardiac output
CI: cardiac index
PAC: pulmonary artery catheter
PICCO: pulse indicator continuous cardiac output
CPB: cardiopulmonary bypass
CABG: coronary artery bypass graft
AD: aortic dissection
EMR: electronic medical record
LVDd: left ventricular diastolic diameter
LAD: left atrial diameter
IVSd: interventricular septum thickness in diastole
LVPWT: left ventricular posterior wall thickness
PASP: pulmonary artery systolic pressure
WBC: white blood cell count
NEU: neutrophil ratio
LYM: lymphocyte ratio
PLT: platelets level
Hb: Hemoglobin
Cr: blood creatinine
BUN: blood urea nitrogen
AST: aspartate aminotransferase
ALT: alanine aminotransferase
TP: total protein
TB: total bilirubin
LDL: low density lipoprotein
CKMB: creatine kinase-MB
T3: triiodothyronine
T4: thyroxine
TSH: thyroid stimulating hormone
BNP: brain natriuretic peptide
Lac: blood lactate
AO: aortic occlusion
UO: urine output
MABP: mean arterial blood pressure
CVP: central venous pressure
MV: mechanical ventilation
AUC: area under curve
ROC: receiver operating characteristic
SABP: systolic artery blood pressure
KNN: k-nearest neighbors
LASSO: least absolute shrinkage and selection operator.
