# Supplementary figures and images for: Prediction of low cardiac output syndrome in patients following cardiac surgery using machine learning
Source: Front Med (Lausanne). 2022 Aug 24;9:973147. doi: 10.3389/fmed.2022.973147 (PMC9448978; doi:10.3389/fmed.2022.973147)

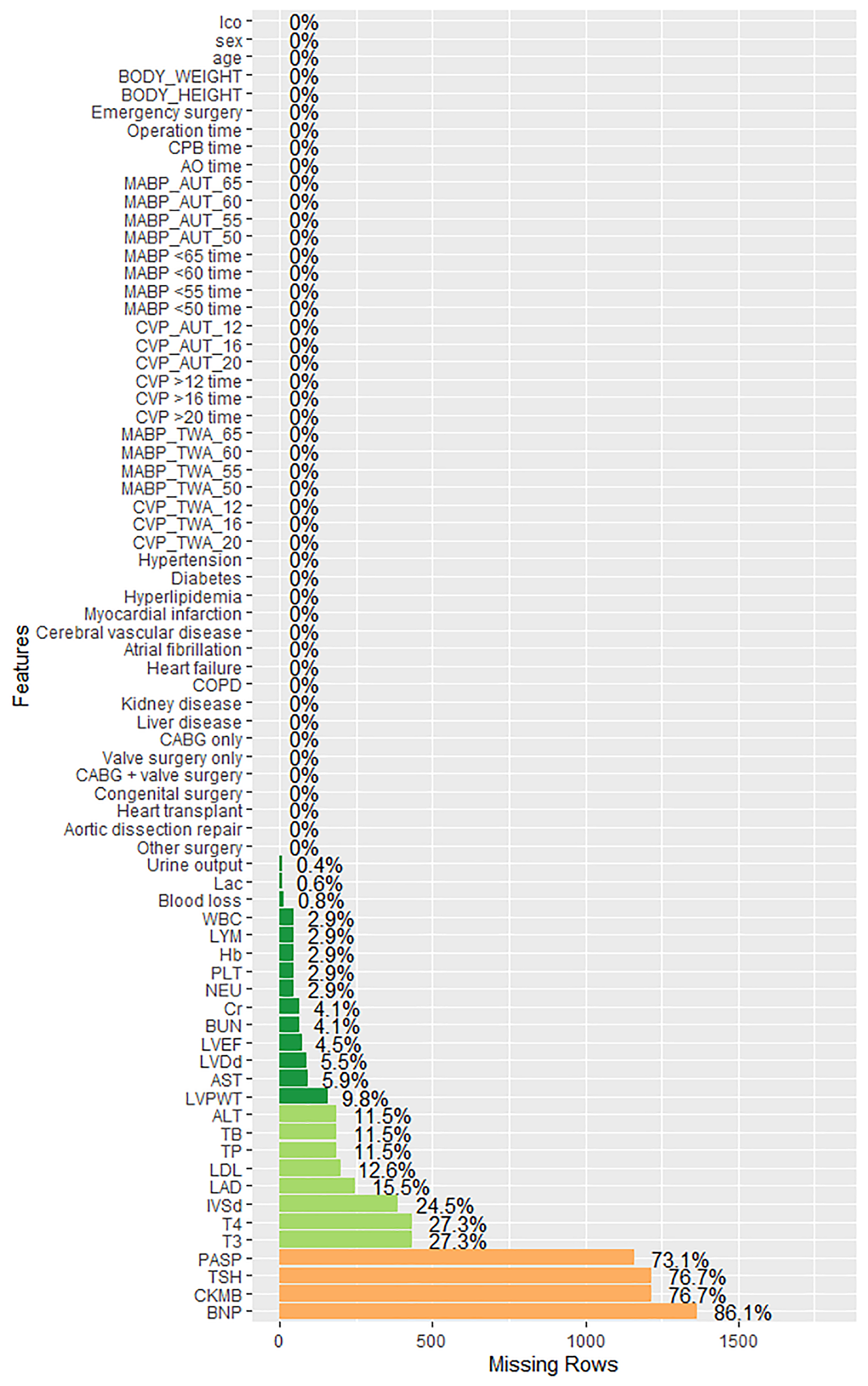

Supplement: Supplementary Figure 1 — Missing value in the study. [file Image_1.JPEG]

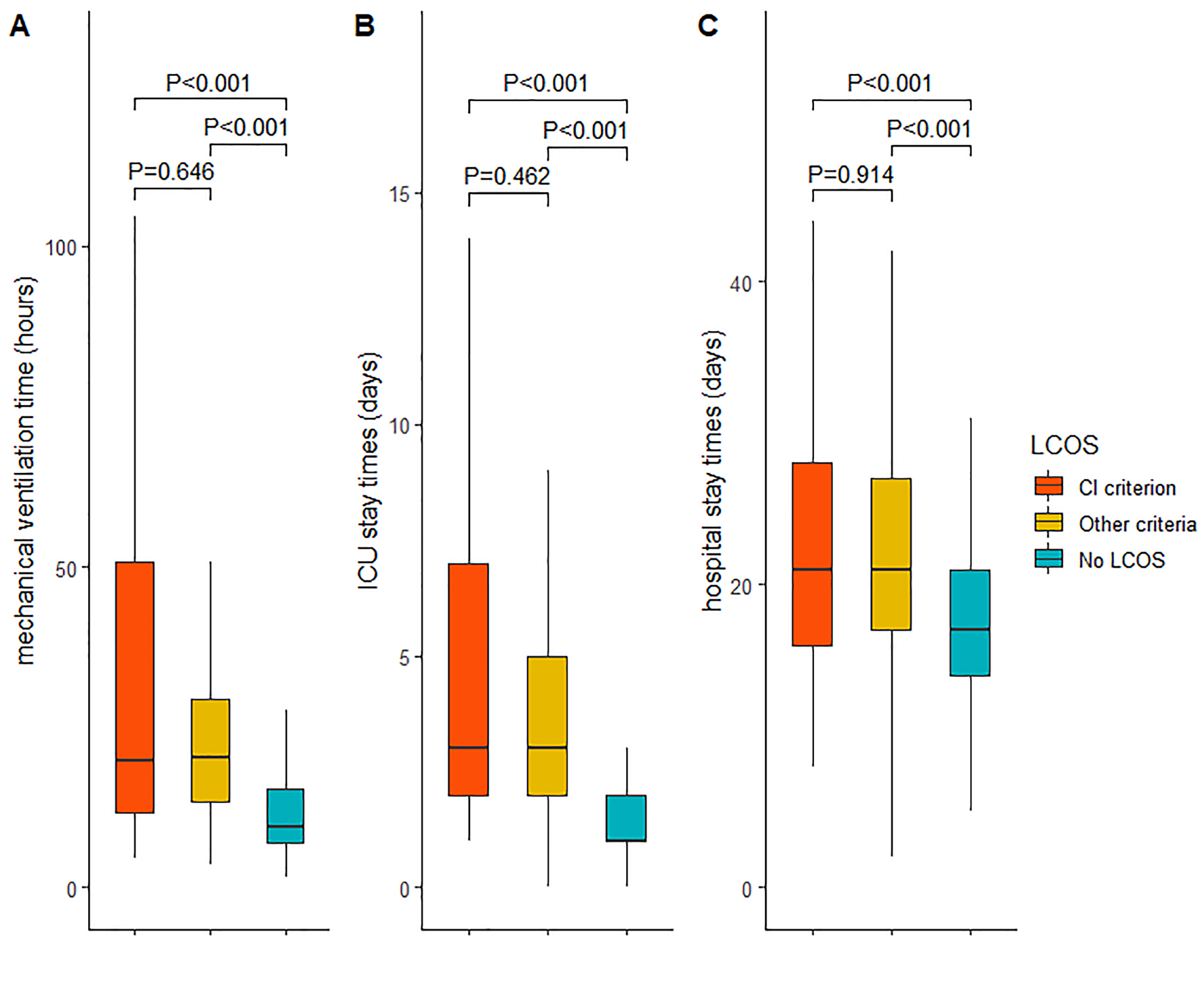

Supplement: Supplementary Figure 2 — Prognosis variables in patients with LCOS diagnosed by CI criterion and other criteria and patients without LCOS, including mechanical ventilation time (A), ICU stay time (B) and hospital stay time (C). [file Image_2.JPEG]

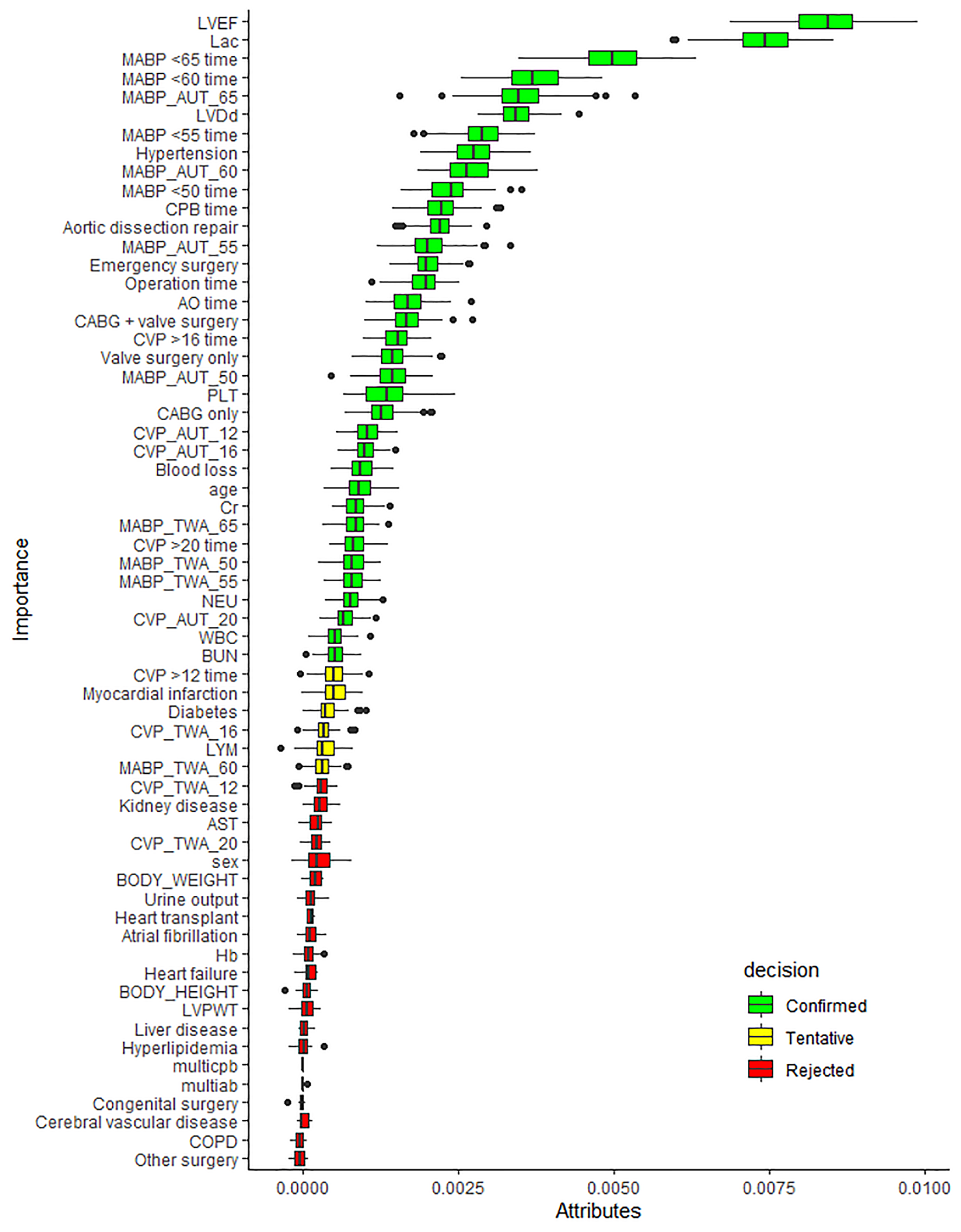

Supplement: Supplementary Figure 3 — Feature importance determined through Boruta algorithm. [file Image_3.JPEG]

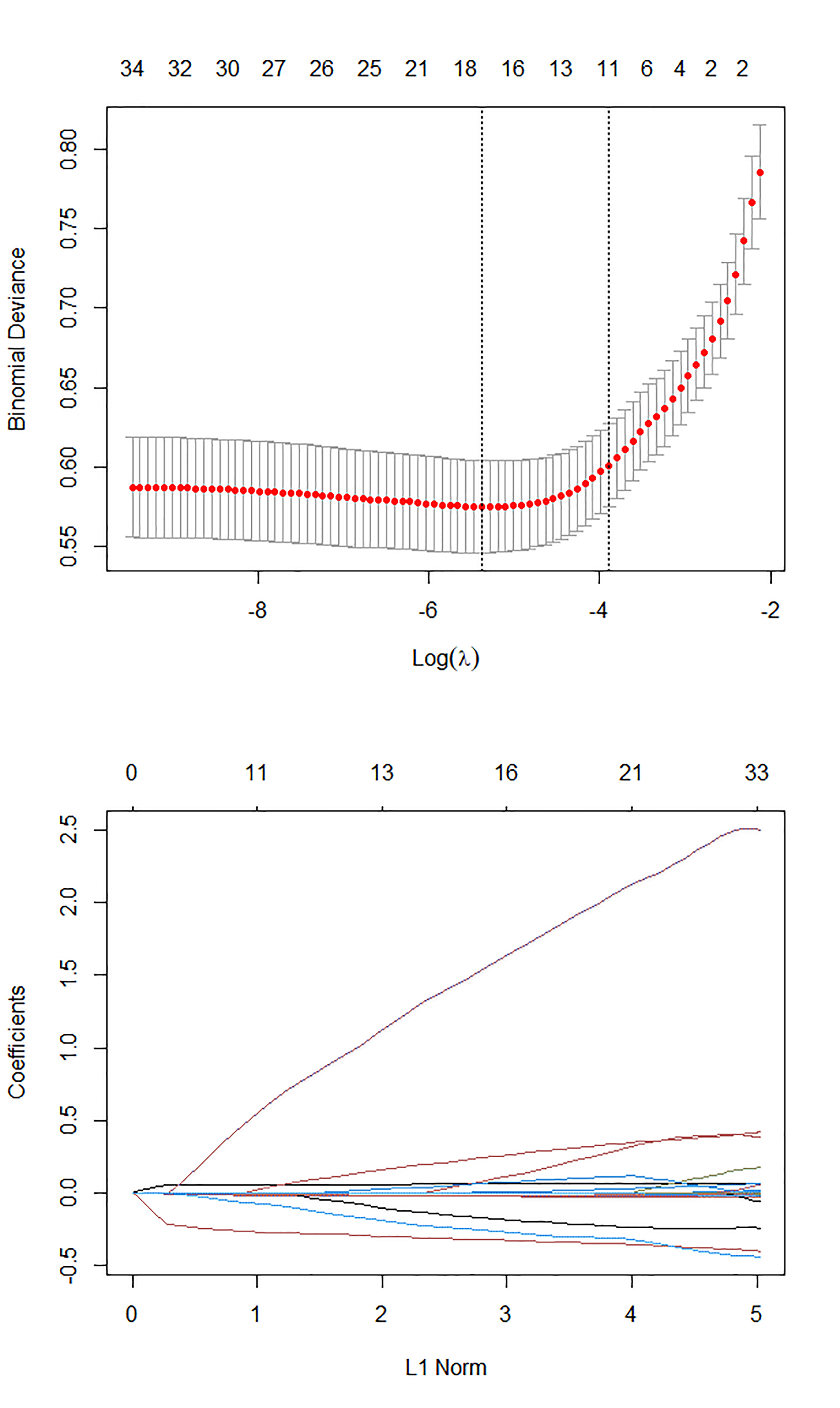

Supplement: Supplementary Figure 4 — Subset feature selection through Lasso regression. [file Image_4.JPEG]
