# Supplementary material for: Prediction of low cardiac output syndrome in patients following cardiac surgery using machine learning
Source: Front Med (Lausanne). 2022 Aug 24;9:973147. doi: 10.3389/fmed.2022.973147 (PMC9448978; doi:10.3389/fmed.2022.973147)
Supplement: Supplementary file 5 [file Table_1.docx]

## Table S1 Codes used for identification of patients with comorbidities

| **Comorbidities** | **ICD-10 code** |
| --- | --- |
| Hypertension | I10, I11, I12, I13, I15 |
| Diabetes | E08, E09, E10, E11, E13 |
| Myocardial infarction | I21, I22 |
| Cerebral vascular disease | I60, I61, I62, I63, I65, I66, I67, I68, I69, G45, G46 |
| Atrial fibrillation | I48 |
| COPD | J40, J41, J42, J43, J44 |
| Heart failure | I50 |
| Liver disease | K70, K71, K72, K73, K74, K75, K76, K77 |
