# Supplementary material for: Prediction of low cardiac output syndrome in patients following cardiac surgery using machine learning
Source: Front Med (Lausanne). 2022 Aug 24;9:973147. doi: 10.3389/fmed.2022.973147 (PMC9448978; doi:10.3389/fmed.2022.973147)
Supplement: Supplementary file 6 [file Table_2.docx]

| **Operation type** | **Patients** | **PAC use** | **Proportion** |
| --- | --- | --- | --- |
| Heart transplant | 18 | 18 | 100.0% |
| CABG + valve surgery | 153 | 70 | 45.8% |
| Congenital surgery | 94 | 41 | 43.6% |
| Valve surgery only | 608 | 170 | 28.0% |
| CABG only | 441 | 69 | 15.6% |
| Other surgery | 164 | 15 | 9.1% |
| Aortic dissection repair | 107 | 3 | 2.8% |

## Table S2 PAC use proportions in different operation type
